# What makes it work? Exploring experiences of patient research partners and researchers involved in a long-term co-creative research collaboration

**DOI:** 10.1186/s40900-020-00207-4

**Published:** 2020-06-19

**Authors:** Emma Hovén, Lars Eriksson, Åsa Månsson D’Souza, Johanna Sörensen, David Hill, Carolin Viklund, Lena Wettergren, Claudia Lampic

**Affiliations:** 1 Department of Women’s and Children’s Health, Karolinska Institutet, SE-171 77 Stockholm, Sweden; 2 Department of Learning, Informatics, Management and Ethics, Karolinska Institutet, SE-171 77 Stockholm, Sweden; 3 School of Health Sciences, City, University of London, London, EC1V 0HB UK; 4 Department of Infectious Diseases, Karolinska University Hospital, SE-141 86 Huddinge, Sweden; 5 Patient research partner, Stockholm, Sweden; 6 Department of Public Health and Caring Sciences, Uppsala University, BMC, Husargatan 3, SE-751 22 Uppsala, Sweden

**Keywords:** Patient and public involvement, Co-creative long-term collaboration, Patient research partners, Cancer research

## Abstract

**Background:**

Exchanging experiences of patient and public involvement (PPI) can bring insights into why, how and when PPI is most effective. The aim of this study was to explore the experiences of patient research partners (PRPs) and researchers engaged in a co-creative long-term collaboration in cancer research.

**Methods:**

The aim and procedures of this study were jointly decided upon by PRPs and researchers. The PRPs included former patients treated for cancer and significant others of the same target group. The participants (11 PRPs, 6 researchers) took part in semi-structured telephone interviews. The interviews were analysed using qualitative content analysis by a researcher who had no prior relationships with the participants.

**Results:**

Five overarching categories were identified: *Reasons for investing in a long-term collaboration*, *Benefits of participating*, *Improving the research*, *Elements of success* and *Challenges and ways to improve*. Reasons for investing in the collaboration included the desire to improve cancer care and to make use of own negative experiences. Benefits of participating included a positive impact on the PRPs’ psychosocial adjustment to the illness. Moreover, the researchers highlighted that working together with the PRPs made the research feel more meaningful. The participants reported that the collaboration improved the relevance and acceptability of the research. Having a shared goal, a clear but yet accommodating structure, as well as an open and trustful working atmosphere were recognised as elements of success. The PRPs furthermore emphasized the importance of seeing that their input mattered. Among the few challenges raised were the distance to the meeting venues for some PRPs and a limited diversity among participants.

**Conclusions:**

This study identified factors essential to researchers and clinicians attempting to engage the public in research. Our results suggest that for successful patient involvement, the purpose and format of the collaboration should be clear to both PRPs and researchers. A clear but yet accommodating structure and keen leadership emerged as key factors to create a sense of stability and a trustful atmosphere. Furthermore, providing regular feedback on how PRPs input is implemented is important for PRPs to stay committed over time.

## Plain English Summary

Exchanging experiences of patient and public involvement (PPI) can bring insights into why, how and when PPI in research brings the greatest benefits. The aim of this study was to explore the experiences of patient research partners (PRPs) and researchers engaged in a co-creative long-term collaboration in cancer research. The PRPs included former patients treated for cancer and significant others of former patients. Participants (11 PRPs, 6 researchers) took part in semi-structured telephone interviews. The analysis identified five overarching categories: *Reasons for investing in a long-term collaboration*, *Benefits of participating*, *Improving the research*, *Elements of success* and *Challenges and ways to improve*. Reasons for investing in the collaboration included the desire to improve care and to make use of own negative experiences. The participants recognised personal benefits of taking part in the collaboration, including that participating had been therapeutic. Positive impact of involvement on the research was also acknowledged. For example, the participants reported that the collaboration made the research more relevant, acceptable and tailored to end users. Among the most prominent elements of success reported by the participants were having a shared goal, a clear but yet accommodating structure, an open and trustful working atmosphere, and seeing that input made a difference. Among the few challenges raised were distance to the meeting venues and limited diversity among the participants. Altogether, the results inform researchers attempting to engage patients in research by describing factors that should be considered when planning and working with PRPs.

## Introduction

Patient and public involvement (PPI) in health and social care research has been reported increasingly over the last decades, and is now regarded as an integral part of good scientific practice [1]. PPI has been defined by the National Institute for Health Research patient and public involvement advisory group, as ‘research being carried out with or by members of the public, rather than to, about or for them’ [2]. The rationale for public involvement in research is that it can enhance the quality, relevance, and uptake of research. More specifically, to incorporate a patient and public perspective may benefit study feasibility and deliverability by ensuring that research objectives, information and measures are relevant and user-friendly [3–6]. Involving patients and the public can also make the research more ethical (e.g., by improving the consent process and the acceptability of the research) and improve dissemination of research to study participants and the wider community [4]. Despite an increasing interest in public involvement, there is still a relatively weak evidence base for the impact of PPI in health research [3, 7–9]. Furthermore, there is currently no consensus available on PPI best practice [10]. While PPI is well established in the United Kingdom (UK), North America and Australia, a recent systematic review of PPI publications in cancer research concluded that other countries are still in its early stages [11]. From a Nordic perspective, researchers have indicated a need for building a better understanding of the concept in order to increase and enhance PPI in health research [12].

Studies on the impact of PPI on patient and public representatives themselves have reported both positive and negative experiences (e.g., [13–16]). Reported benefits of patient involvement include an enhanced understanding of research [14] and a positive impact on one’s health and well-being, e.g., by coming to terms with one’s illness [13, 14]. Reported negative experiences of PPI relates to being overlooked and underused, feeling overwhelmed and not having been informed about the objectives of involvement activities [13, 14, 16].

Long-term collaboration with patient representatives has been proposed to increase the relevance, quality, and validity of eHealth interventions [17, 18]. Still, patient involvement in eHealth intervention research has, with a few exceptions [19], been limited to the consultation level and involved patient representatives only on single occasions [20, 21]. Exchanging experiences of PPI and getting feedback from patients and researchers can bring insights into why, how and when patient and/or public involvement in research brings the greatest benefits. Such evaluation efforts are encouraged as this “can improve the involvement process, spur mutual learning, and change researchers’ mindsets and future practice” [22]. To our knowledge, studies that take into account the perspectives and experiences of both patient research partners (PRPs) and researchers involved in long-term collaborative research are scarce. Furthermore, there is a lack of studies on patient involvement in health research that are co-conceived and co-conducted with PRPs. Thus, the aim of this study was to explore the experiences of PRPs and researchers engaged in a co-creative long-term collaboration in cancer research (the Fex-Can project, see below). It was of particular interest to capture their reflections on the impact of patient involvement and to evaluate the collaboration by including PRPs’ and researchers’ views on the forms for collaboration. In doing so, we aimed to advance knowledge about what patient involvement works, in what context and why.

### Patient involvement in the fertility and sexuality following cancer (Fex-can) project

The present study was conducted in the context of the Fex-Can project. Besides investigating the prevalence of fertility distress and sexual dysfunction in young adults with cancer during the first 5 years following diagnosis, (Wettergren L, Ljungman L, Micaux Obol C, Eriksson L, Lampic C: Sexual dysfunction and fertility-related distress in young adults with cancer over 5 years following diagnosis: study protocol of the Fex-can cohort study, unpublished) the project aims to test the effect of a web-based psycho-educational intervention on these problem areas in a randomized controlled trial (RCT) [23]. The collaboration between researchers and PRPs is a core feature of the Fex-Can project. This collaboration commenced in 2014 and was planned to continue over the five-year course of the intervention development and evaluation. The present study focuses on the experiences of the PRPs and researchers involved in this long-term collaboration.

#### Patient research partners (PRPs)

Ten former patients treated for cancer and two significant others of the same target group were recruited from a previous study investigating sexuality and fertility among survivors of childhood cancer [24], and through cancer nurse navigators at a university hospital. A more detailed description of the recruitment of the PRPs has been reported previously [25]. All PRPs agreed to participate in a five-year-long collaboration within the Fex-Can project. The number recruited was based on the belief of a suitable group size of at least 5–8 participants at gatherings, taking into account absence at occasional meetings as well as attrition (dropout) from the project. All PRPs were born in Sweden, they lived in different parts of the country and the majority had a university degree. They worked or studied in various fields but none of them had a background in healthcare or health-related research. The former patients, six women and four men, had been diagnosed in adolescence or young adulthood and were aged 20–41 at recruitment. The significant others were two mothers of teenagers who had undergone cancer treatment. During the collaboration the PRPs had been reimbursed for travel expenses and accommodation, and were paid for their participation in project meetings and time working with assignments*.*

#### Research group

The group included senior and junior researchers with academic backgrounds in nursing, medicine, psychology and psychiatry. Clinical backgrounds of the research group members included primary care, psychotherapy, and counselling in cancer care and sexually transmissible infections. The research group members are from now on referred to as ‘researchers’*.*

The forms for collaboration, including the length, frequency and structure of meetings, were agreed upon during the first meeting and were revisited on subsequent meetings. The collaboration mainly took place in the form of half-day meetings on a regular basis, while communication between these meetings was carried out by email. An overview of the collaboration in the Fex-Can project is presented in Table 1. The research group was responsible for managing the collaboration with the PRPs, including strategic and logistic planning of meetings with the PRPs. The meetings were scheduled to ensure that the PRPs always were in majority at the gatherings that included plenary and small group discussions as well as individual assignments. The working relationship has covered different activities and included the development of the web-based psycho-educational intervention using a co-creative design. The process was performed according to the design steps in the holistic framework by van Gemert-Pijnen and colleagues [26]. For example*,* the PRPs were presented with suggestions and samples of intervention material, which were discussed in detail and revised and refined for presentation at subsequent meetings. Aspects regarding e.g., graphical design of the website, study procedure and design, and outcome measures were also discussed and refined in cycles. A more detailed description of the PRP’s impact on the content, system, and service quality of the planned intervention can be found in a previous publication by Winterling and colleagues [25]. In addition, some PRPs contributed to the web-based intervention with their personal stories, and one PRP participated as moderator of the discussion forum during the RCT.

**Table 1:** Overview of first four years of patient involvement in the Fex-Can project

Patient involvement meetings	PRPs	Researchers^a^
Half-day meetings on Saturdays, including lunch	10 former patients 2 mothers of former patients	9 senior researchers, postdocs, doctoral students
2–3 meetings yearly, total of 10 meetings	Median number PRPs at each meeting: 7	Median number researchers at each meeting^b^: 5
	Each PRP attended a median of 5 meetings	Each researcher attended a median of 7,5 meetings

PRPs: Patient research partners

^a^ The composition and number of research group members varied over the collaboration period

^b^ including two senior researchers (project leaders) who both attended all but one meeting

## Methods

### Design

The aim and procedures of the present study were jointly decided at a scheduled patient involvement meeting in March 2018, where five of the PRPs and three of the researchers participated. Based on a discussion at a previous patient involvement meeting, the meeting included a work-shop on the possibility to share our common experiences of the research collaboration at an international scientific conference. In an open and creative discussion, the group decided to conduct an interview study to gain deeper understanding of the motivations for and experiences of the long-term collaboration among all involved PRPs and researchers.

The reporting abides by the Guidance for Reporting Involvement of Patients and the Public (GRIPP2) (see additional file) [27].

### Participants

Of the 12 PRPs, one chose not to participate in the interview study due to personal circumstances, leaving 11 participating PRPs (4 men, 7 women; 9 former patients and 2 mothers of former patients). Of a total of nine researchers who had been involved in the collaboration with the PRPs, those six persons who had participated in ≥4 patient involvement meetings were approached for the present interview study and all agreed to participate (1 man, 5 women).

### Data collection

The researchers and PRPs at the above-mentioned meeting developed an interview guide covering topics identified as relevant (Table 2). In order to optimize free expression of experiences, it was agreed that all interviews should be conducted by the PRPs, and four of those present at the meeting volunteered to this task. The group decided that the researchers should provide the necessary structure. This entailed mailing information about the study aim, procedures and interview questions to all PRPs and researchers, and explaining the voluntary nature of participation. The researchers also provided the four PRPs who would be conducting the interviews with a work plan with clear instructions, contact information to assigned interviewees, as well as digital tools for recording of telephone interviews. This study was part of the Fex-Can project, which was provided ethical approval in November 2013 by the Swedish Ethical Review Authority (reference number: 2013/1746–31/4). All participants provided verbal informed consent to participate in this study.

**Table 2:** Interview guide

Why did you join the collaboration?	
How have you participated?	
What does the collaboration mean to you?	
How have patient research partners contributed to the project?	
What do you think works in the collaboration?	
Why do you think it works?	
What could work better or be done differently?	

The participants took part in semi-structured telephone interviews. Thirteen individual interviews and two dyadic interviews, in which two PRPs interviewed each other on the same occasion, were conducted. With the exception of two interviews, all were conducted by the four PRPs who volunteered to this task. For practical reasons, two individual interviews were performed by a psychologist in the research team who had no prior relationship with the participants. The interviews were carried out between April and June 2018. On average they were 20 min long, and all were audio-recorded and transcribed verbatim. The interviewers also took field notes during the interview.

### Analysis

The PRPs and researchers at the above-mentioned meeting decided that the researchers should have the main responsibility for the analysis of the interview data. The first author (EH), who was not part of the research group and who had no relationships with the participants (PRPs or researchers) prior to the commencement of the study, conducted the analysis. The transcripts were analysed using qualitative content analysis, as described by Graneheim and Lundman [28]. The analysis followed the following steps: (1) each transcript was read to gain an overall understanding of its context and content; (2) meaning units were identified, defined as sections that shared a common meaning and context related to thoughts on the collaboration; and (3) meaning units were assigned a descriptive code, defined as a label of the meaning unit’s content. Codes were transferred into sub-categories which were sorted into categories, characterised as a group of content that shares a commonality. An example of the analysis process is shown in Table 3.

**Table 3:** Example of the content analysis process

Meaning unit	Code	Sub-category	Category
It has been good that the researchers have been there to develop a framework and to move the dialogue on.	A thought-through and clear structure	Clear yet accommodating structure	Elements of success
I think it’s important that we have this openness and freedom. That you, that everyone feels that you contribute when you can, and as much as you can, without demanding too much from the individual.	Voluntary participation		
It’s exciting that there are so many people that can think and feel so much. Still, I do think we can have rather different views. It often goes rather “smoothly” but it can also be a bit, that you have different opinions about things, and I think it’s fascinating that it still works so well.	Different views are expressed and handled	Open and trustworthy atmosphere	
Well, we have this follow-up every six months to see how things have been going, and the issues that you have suggested to maybe change or keep, that this has actually been done. So, it’s nice to see that what you say at the gatherings actually helps.	Feedback on how the input is used	Input made a difference	

The first and last two authors held repeated meetings during the course of the analysis, where they discussed the identified meaning units, codes, and overarching sub-categories and categories. When all interviews were analysed, one of the PRPs who performed some of the interviews (the third author) was invited to review the findings and share her reflections on whether the interviews were thoroughly covered by the findings. At this point, all remaining authors judged that the findings thoroughly portrayed the data.

## Results

The analysis resulted in five overarching categories: *Reasons for investing in a long-term collaboration, Benefits of participating, Improving the research, Elements of success* and *Challenges and ways to improve*. The categories and sub-categories are presented in Table 4. In the following results presentation, the term ‘participants’ is used to denote experiences described by both PRPs and researchers. All participants were provided with an individual study number and each quote is marked with the participant’s role and number, e.g. PRP #5.

**Table 4:** Categories and sub-categories of participants’ experiences of the co-creative collaboration

Category	Sub-category
Reasons for investing in a long-term collaboration	Negative experiences of information and care
	Desire to improve cancer care
	Engaging topics
	Chance to get answers to own questions
	The form for the collaboration attracted interest
Benefits of participating	Participation as a kind of therapy
	An educational and fun activity
	My work feels relevant and meaningful
Improving the research	Contributes to making the research project relevant, acceptable and tailored
	A more thought-through research process
	Indicates quality of the project
Elements of success	Engaged and committed participants
	Strive towards a common goal
	Clear yet accommodating structure
	Open and trustworthy atmosphere
	Input made a difference
	A diverse group
	Nice and fun to meet
	Good leadership
Challenges and ways to improve	Greater diversity of group members
	Alternative structure of collaboration to advance progress
	Distance to meeting venue as an obstacle

### Reasons for investing in a long-term collaboration

From the perspective of both PRPs and researchers, the main reason for investing in the long-term collaboration was the desire to improve cancer care. Participants described having the general idea that “when one has the chance to help others one should”, and that investing in this collaboration would mean that one’s own experiences will be utilized. PRPs emphasized that their own negative experiences of care and support after the cancer diagnosis motivated them to join the collaboration in the first place, offering an opportunity to voice and address perceived shortcomings of care.

*“I wanted to contribute to help improving the care of future patients. It was something I could easily do to help, where I together with the other PRPs have unique competence.”* (PRP #5).

Furthermore, PRPs described that the collaboration offered an opportunity to get answers to questions about the care and support they had received, and that they still felt were unanswered. Another reason, reported by both PRPs and researchers, was that the collaboration concerned issues that they found engaging and interesting. A specific factor that motivated PRPs to sign up for the long-term collaboration was related to how the arrangement of the collaborative work and their role in it were presented when they were invited to the project. In particular, they mentioned that the collaboration sounded like a fun activity that also provided an opportunity to meet others with a history of cancer.

### Benefits of participating

The participants emphasized that being involved in this collaboration had provided personal benefits. Gaining such benefits was described as unexpected by some of the PRPs. They described the collaboration as a kind of therapy that helped them in their adjustment and rehabilitation. Getting something back from being involved in the research project was described by PRPs as a source for continued motivation and engagement. The collaboration provided a setting where they were able to give voice to their feelings and concerns, and exchange experiences with others who had been affected by cancer. The collaboration made them realize that their feelings, concerns and problems were not unique, but rather something that many do experience, even persons without a history of cancer. Furthermore, to voice their experiences in the group gave some the confidence to share experiences also outside the group. Another therapeutic element for the PRPs was learning that there are sources of help available for certain problems and/or concerns.

*“I felt very alone with this concern, but as soon as you started to open up you realized that there were others who had the same problem but not related to cancer.”* (PRP #9).

*“For me, it has been, it has actually been sort of a rehabilitation journey.”* (PRP #3).

The participants described the collaboration as educational and fun. They expressed that they had learned a lot from the collaboration, for example about how PPI in research actually works and what it entails. They also emphasized that they had enjoyed the collaboration and that they often felt happy and energized after the meetings. Furthermore, the PRPs described that they perceived their contributions as important and meaningful. They mentioned that the collaboration had meant a lot them, and that it felt good to contribute to this research project that aimed to make a difference for future patients.

*“Yes, it has meant very much to feel that my experiences can be important to someone else. And that it actually, the things that I felt were bad, that I get the opportunity to participate and tell this to someone so that it can be better for someone else.”* (PRP #2).

Moreover, the researchers described the collaboration as very meaningful, personally and professionally. Working together with the PRPs made them feel that they were doing an important and relevant job, which motivated them in their daily work within this research project.

*“Well, it’s always fun to meet people with different experiences and, like, interact. I find it enriching both for the research and for my work, and for me personally as well.”* (Researcher #3).

### Improving the research

Both PRPs and researchers described that involving patient representatives was of great significance for the research project. It was emphasized that the collaboration contributed to making the research more relevant, acceptable and tailored to the target population. Specifically, insights and perspectives of PRPs were important for the development of the format and content of the intervention. Moreover, the participants expressed that the inclusion of PRPs helped incorporating a perspective from outside the academia and research system.

*“Well, it’s about contributing with this insider perspective or something. That they actually have their own lived experience of what we are trying to understand from the outside*.” (Researcher #3).

*“It’s a way to, based on your own experiences, find out how one can somehow improve the portal [the web-based program] for example, so that it doesn’t end up so, so confined or too research, research-focused, but rather adapted to the actual issues one is confronted with. / … / So I believe, adaptation based on relevance rather than theory./ … / I think it’s hard to relate to what it is one must face if you haven’t been through it yourself.”* (PRP #1).

It was described that more thought was given to the research process thanks to the collaboration. For example, researchers described that having research partners involved in the project reminded them of the end goal of the project in every step of the way.

*“So that we don’t risk getting stuck in a sort of ‘research mode’, that it’s exciting to explore things. But here the aim is to improve the care / … / To constantly remind us that this is the end goal so that we don’t lose sight of that focus.”* (Researcher #6).

Furthermore, the participants pointed out that having established a collaboration with PRPs is an indicator of quality of research, i.e., that patient involvement in itself is a mark of quality of the research.

*“We [the PRPs] have like significance to, for the research project. Like a mark of quality, increased, yes, simply an increased quality.”* (PRP #4).

### Elements of success

The participants had a genuine interest in the topic of the research project and were engaged in whatever task was at hand. This commitment to the collaboration among both the researchers and PRPs was described as a key factor for why the collaboration had worked well. Moreover, having a common goal that all strived towards was described as essential for sustaining the long-term collaboration. To have a common goal that was clear to all was regarded as the main link between the participants.

*“Well, we do have, I think it’s like everyone has a common cause that binds us together.”* (PRP #6).

Another element of success concerned the clear but yet accommodating structure of the collaboration. One important factor was the format for meetings and assignments, which was open for discussion, and that participants generally accepted and found practical. The clear distribution of roles between PRPs and researchers, and the structure of providing recurrent feedback to PRPs on the progress of the project and the implementation of their input, gave a sense of stability. Another important factor was the financial compensation to PRPs for lost working hours and travel expenses. Participants also acknowledged the value of participation being voluntary, and appreciated that one could invest as much as one wanted in the collaboration, and be free to attend or be absent at gatherings depending on availability.

*“I think it works really well because, partly because it’s once per semester. And that it’s like a whole day so that it gets, or a full day, a few hours. So that it gets pretty intense so, so that you really are immersed in it.”* (PRP #2).

*“Well, I think it’s the clarity around the framework, assignments, it’s clear. We provide the invitation, the framework is clear, there’s an agenda, there is this lunch, it’s sufficiently structured. And that means we all trust in and feel confident to continue this project with a very clear framework and build on what we have said before. That you believe what you have to say, or what you yourself are interested in or feel is important, will be made use of, because then there is reason to come.”* (Researcher #4).

The collaboration was characterised by an open and trusting atmosphere, which was described as a key element to a successful and sustainable collaboration. The participants pointed out that all were encouraged to share their experiences and opinions, regardless if positive or negative. When differing views were expressed these were discussed in a respectful manner. The PRPs furthermore recognized that they were treated as equals, which contributed to an atmosphere of mutual respect, empathy and trust.

*“And then, when we meet, I think it works very well because it’s an open atmosphere, and everyone is very understanding and empathetic. So you can, you don’t have to be afraid to say what you think and feel, but it’s a very understanding atmosphere.”* (PRP #2).

The PRPs found it important to see that their input was incorporated in the project, i.e. that their opinions and suggestions actually made a difference. Receiving feedback on how their input was utilized contributed to a sense of meaningfulness.

*“And you really feel that those who lead this project listen to you and acknowledge what we say, regardless of whether it’s just one person saying it, they really take it into account. And that, for example, the texts are revised based on that.”* (PRP #2).

Participants described the value of diversity in the group of PRPs, including both patient representatives and significant others who differed in age, cancer experience and areas of residency. Furthermore, participants mentioned that the size of the group was suitable. While it was large enough to allow for different opinions and experiences to be raised, it was small enough for everyone to get to know each other and have their say. Participants also mentioned that a smaller group would have made the gatherings dependent on everyone showing up.

*“Well, there’s a variation of ages and different diagnoses and everything, so I think, and from different areas in the country, and I think that has also been positive. So, not everyone has the same background, in any way, not in terms of disease characteristics either.”* (PRP #7).

Moreover, when participants talked about what makes the collaboration work well and keep participants committed, they expressed that the group members enjoyed each other’s company and that the meetings were enjoyable.

*“It’s partly a coincidence, that it ended up being nice people that have met, but I also think that the individuals, that we like being together, and that’s also what makes, drives our collaboration and makes everyone want, want to be part of this and return.”* (Researcher #1).

*“Well, it has been a nice atmosphere, like in the group and so. / … / we usually have some food and so on at the meetings. So, that definitely contributes to a more relaxed atmosphere rather than becoming, like kind of stiff or something.”* (PRP #9).

The PRPs explained how the leadership had been of great importance for the successful collaboration. The leadership was described as knowledgeable and keen. It was mentioned that the meetings were perceived as meaningful because the researchers had led the meetings with such professionalism, e.g. by facilitating the discussions on the focal topics.

*“And then it doesn’t get like messy, but the researchers keep it [discussions at patient involvement meetings] under control so it goes smoothly, and I think it’s done very professionally. It makes you feel safe. / ... / And then, you feel important and the researchers are very careful about showing that our presence and input are important. You really get that feeling.”* (PRP #6).

### Challenges and ways to improve

The participants described that the diversity of the group could have been better. Group members of differing ethnicities, backgrounds and educational experiences might have brought up different perspectives and important experiences. Moreover, it was mentioned that the PRPs contributed with important experiences and opinions to the project, but few actually expressed major problems with the issues that the intervention targeted.

*“Sure, the ideal would have been to find persons who were committed and also had a lot of problems.”* (Researcher #5).

*“Possibly, if you want more different ethnicities or something …*” (PRP #9).

Alternative ways to organize the collaboration were discussed in terms of how this might have advanced the progress of the project. Suggestions included having more frequent gatherings, having online meetings alongside gatherings, and having the opportunity to work with material from home. The researchers expressed ideas on how to engage PRPs in other ways and in other tasks. For example, having PRPs work part-time in the project, and giving them the chance to freely come up with ideas on how the research project should evolve. The participants mentioned that a potential drawback of long-term collaborations is the risk that members will become less objective and less critical over time. Furthermore, the participants described how distance to the meeting venue was an obstacle, and suggested that the time for meetings should be prolonged since some travelled quite far for the half-day meetings.

*“Now it may be included somehow in this latest project [the present study] but, well, one could have let you [PRPs] control the agenda even more, regarding what we should address, like what’s important.”* (Researcher #3).

*“You could have virtual meetings, Skype or something. It would make it a little easier sometimes because ... when I attend a meeting, it takes like the whole day, even if it [the meeting] is only half a day.”* (PRP #8).

## Discussion

This exploration of PRPs’ and researchers’ experiences of being involved in a four-year co-creative research collaboration offers unique insights into patient involvement in eHealth intervention research, a relatively unexplored area. The study identifies factors important to researchers and clinicians attempting to engage patients in research. A variety of reasons for investing in a long-term collaboration were described by the participants. These reasons included the desire to improve cancer care, getting the opportunity to make use of own negative experiences and to work with engaging topics. The participants furthermore recognised personal benefits of taking part in the collaboration and also acknowledged positive impact of involvement on the research, such as making the research more relevant, acceptable and tailored to end users. A main finding of this study concerns the elements of what makes a long-term research collaboration with patient involvement successful according to the participants. Among the most prominent elements were striving towards a shared goal, a structure of the collaboration that was clear but yet accommodating, a working atmosphere characterized by openness and trust, and seeing that input made a difference, i.e. that ideas and comments were implemented in the research. The participants expressed overwhelmingly positive views of the collaboration while few negative experiences were reported.

To our knowledge, the present study is the first to offer a comprehensive investigation of both PRPs’ and researchers’ experiences of a long-term co-creative research collaboration in eHealth intervention research. In Sweden, patient involvement in research is emerging with increasing numbers of researchers attempting to engage patients in their work. By sharing the experiences of PRPs and researchers involved in the Fex-Can collaboration we hope to provide guidance on how to set up and enhance patient involvement in health research. Furthermore, while studies on experiences of patient involvement in cancer research do exist [11], we are not aware of any such studies that have been conceived and conducted together with PRPs. To truly explore the potential of patient involvement, PRPs should indeed be involved throughout the research process.

Increased understanding of PRPs’ motives for getting involved in research and their expectations of such involvement can inform researchers about ways to recruit patient representatives and encourage their involvement. The participants in the present study expressed altruistic motivations for getting involved. For example, participants described that they were motivated to sign up for the collaboration since it provided an opportunity both to improve cancer care for future patients and to make use of own negative experiences. This finding mirrors results of studies on users and carers engaged in practitioner education [29], in the National Cancer Research Network in the UK [15], and in a variety of diagnostic research specialties in the UK [14]. However, contrasting the study by Ashcroft and colleagues [14], being motivated to join for personal development reasons or for financial gains was not reported by the present study participants. Our findings suggest that patient representatives may not be fully aware at the outset that they can gain personal benefits from being involved as PRPs. Some participants reported that the proposed form for collaboration seemed to be fun and offered an opportunity to meet others with a history of cancer, which motivated them to get involved. These findings emphasize the importance of presenting the concept of patient involvement, and the proposed format and purpose for the research collaboration, in a clear and engaging manner. We recommend that the forms for collaboration are discussed early on, in the first phase of the collaboration, since we believe that this is a key factor for successful patient involvement. Furthermore, such discussions can be a good illustration of a working relationship on equal grounds. This in turn can inspire both PRPs and researchers, and open up for development of both the content of the research as well as suitable and feasible forms and structure of the collaboration.

The participants in the study described various positive impacts of patient involvement on the research project. The involvement of the PRPs was perceived to contribute to creating a more thought-through research process, constantly reminding the researchers of the end-goal of the collaborative work. In line with what has been shown in the literature on PPI in a range of health and social care research [3, 5, 9, 30], the participants in this long-term collaboration acknowledged that the PRPs contributed by assessing the appropriateness of research questions, study design, working material and outcome measures. Furthermore, descriptions of the involvement of the PRPs concerned how it facilitated the development of a relevant and acceptable intervention tailored to young adults with cancer. The concrete outcomes of the PRPs’ impact on the development of the intervention have been described previously [25]. A feasibility study of this intervention showed promising results in terms of demand, acceptability, preliminary efficacy, and functionality [31]. The effectiveness of the interventions is to be tested in a RCT trial [23].

Besides a positive impact on the research process, both PRPs and researchers recognised personal benefits of being involved in a co-production of research. Personal positive outcomes were reflected in PRPs’ statements of therapeutic benefits, feeling useful and enjoyment of patient involvement activities. In accordance with previous studies from the UK on involvement across mental health trusts [16], in a variety of diagnostic research specialties [14], and in a cancer setting specifically [15], the PRPs in this study described that the involvement had provided opportunities for personal development and empowerment. The findings suggest that the involvement provided them insights and means of meaning-making. The psychological, social and intellectual personal benefits reported by the PRPs provide a nuanced understanding of the wider implications of involvement. To feel useful has previously been reported as a motivation for getting involved in research [14]. In line with this, the PRPs in the present study mentioned their experiences of making important and meaningful contributions to something useful for others as positive personal outcomes. Participants described that it felt good that their experiences and opinions were made use of, which can be seen as an important motivation for participants to stay involved over a long period of time*.* Furthermore, consistent with previous studies conducted in the UK with differing approaches of PPI [32, 33], our results show that the researchers felt that involving PRPs was valuable to them personally, beyond the impact on the research process. They described that collaborating with the PRPs was inspiring and contributed to the feeling of doing important work. Overall, the experience of patient involvement was seen as a positive part of their daily work. In view of previous reports showing negative experiences of PPI among researchers [33], the finding that no strains or negative experiences were reported by the researchers involved in this four-year collaboration is notable and encouraging.

Our findings identified a range of factors associated with effective patient involvement. We believe that these factors are of particular importance for researchers in countries like Sweden where the involvement of patient representatives as collaborators in research is expanding, but where there is a need to enhance the knowledge of how to best work with PRPs [12, 34]. For increased knowledge of how to successfully involve patients in research, initiatives should come from the research community, health authorities as well as patient organisations [12]. Internationally, the UK has been leading in developing such initiatives for improving PPI in health research. Examples include the government-funded programme INVOLVE [35] and the James Lind Alliance [36], which both work to bring together expertise, insight and experience of PPI to advance the process by which research is identified, prioritised, and conducted. The results of this study showed that one key factor for effective patient involvement was having a clear but accommodating structure of the collaboration. The participants expressed that the roles of the PRPs and the researchers were clear to them, which is an important finding since the lack of clarity regarding expectations and roles has been identified as a barrier to PPI [14]. While the participants mentioned alternative ways to work together, such as online meetings, it is clear from our findings that both the PRPs and the researchers valued having face-to-face meetings, which allowed for social interactions and the building of relationships. Furthermore, our findings highlight the importance of acknowledging the input from PRPs by providing feedback on the value of their contribution, which previously has been shown to be important in maintaining motivation and confidence among PRPs [37]. We recommend that researchers engaging in PPI set in place a specific system to ensure that PRPs are given recurrent feedback on how the research advances and how their input is implemented in the research.

It should be noted that several of the elements that we identified as important for successful collaboration may be a result from the long-term nature of our collaboration, with the same participants having been involved for several years. This is in line with the National Institute for Health Research funded RAPPORT study [38], which emphasizes establishment of relationships maintained over time as a key characteristic for effective PPI that goes beyond the consultation level. The PRPs in the present study emphasized the importance of having been treated as equals for the success of creating an atmosphere of mutual respect, empathy and trust, which is something that may take time to establish. Indeed, it has been suggested that participation of PRPs on an equal footing is a key for facilitating meaningful interactions and knowledge exchange [39].

Despite probing questions about negative experiences of the collaboration, very few negative comments were made. Among the few challenges mentioned were distance to the meeting venues and a lack of diversity in terms of race, education and perceived problems related to the focus of the Fex-Can project. The group’s composition reflects a critical remark pointed out in a recent systematic review on PPI in cancer research, namely the apparent over-representation of well-educated female participants from ethnic majority groups among public and patient representatives [11]. It is clear that further work is needed on how to engage under-represented groups in PPI in health research. We recommend that future studies work actively towards achieving diversity among PRPs, using innovative strategies for recruitment. Different approaches to attract and connect with new members should be tested. One example we have discussed within our collaboration is to have experienced PRPs act as mentors for new PRPs.

### Strengths and limitations

A main strength of the present study is that the study was conceived, conducted and disseminated by a group of five PRPs and three researchers. The preliminary results of the study were jointly compiled by this group and presented at the International Society of Quality of Life (ISOQOL) conference in 2018 [40]. We believe that the active involvement of PRPs in designing the study, collecting the data, reviewing the findings, and disseminating the results increases the authenticity and trustworthiness of the findings. Based on their significant contributions, all PRPs were offered co-authorship on this paper. Another strength of this study is that the interviews yielded data on a diverse range of views and experiences of the collaboration. Furthermore, we believe that the credibility of the results is strengthened by the fact that the analysis was led by an external researcher with no previous involvement in the Fex-Can project. Still, some limitations should be considered when interpreting the results of this study. Even though the participants emphasized that the collaboration was characterized by an open atmosphere where both positive and negative comments were welcomed, we cannot rule out the possibility that some may have felt reluctant to speak critically about the collaboration during the interviews. The potential influence of power dynamics on patient involvement is a well-known challenge [41]. One strategy to counter this in the present project was to ensure that PRPs were in the majority at all meetings. Furthermore, the study interviews were conducted by PRPs with the ambition to create an atmosphere where the participants would feel comfortable in expressing their feelings and thoughts about the collaboration. However, we must acknowledge the risk of researchers not feeling comfortable to express negative thoughts about the collaboration with PRPs.

## Conclusions

By taking into account both the PRPs’ and researchers’ experiences of being involved in a four-year co-creative research collaboration, our results inform practice by identifying factors that facilitate successful and meaningful patient involvement in a research setting. This study highlights that the purpose and format of the collaboration should be clear both with regard to content, and the roles and expectations of PRPs and researchers. A clear but yet accommodating structure and a keen leadership emerged as key factors in creating a sense of stability and a trustful atmosphere. Furthermore, the results of this study underscore that providing regular feedback on how PRPs’ input is implemented in the research increases the likelihood of PRPs remaining committed in a long-term research collaboration. A central aspect of the present study is that it was conceived, designed and conducted in collaboration with PRPs. We encourage future studies of patient involvement to be conducted in a similar co-creative manner, extending the work with PRPs to include all stages in a research project.

## Data Availability

The datasets used and analysed during the current study are available from the corresponding author on reasonable request.

## Abbreviations

Fex-Can: Fertility and sexuality following cancer
GRIPP2: Guidance for Reporting Involvement of Patients and the Public 2
ISOQOL: International Society of Quality of Life
PPI: Public patient involvement
PRP: Patient research partner
RCT: Randomized controlled trial
UK: United Kingdom
